# *mecA*-related structure in methicillin-resistant coagulase-negative staphylococci from street food in Taiwan

**DOI:** 10.1038/srep42205

**Published:** 2017-02-09

**Authors:** Tsung-Ying Yang, Wei-Wen Hung, Lin Lin, Wei-Chun Hung, Sung-Pin Tseng

**Affiliations:** 1 Department of Medical Laboratory Science and Biotechnology, College of Health Sciences, Kaohsiung Medical University, Kaohsiung, Taiwan; 2 Division of Endocrinology and Metabolism, Department of Internal Medicine, Kaohsiung Medical University Hospital, Kaohsiung Medical University, Kaohsiung, Taiwan; 3 Department of Culinary Art, I-Shou University, Kaohsiung, Taiwan; 4 Department of Microbiology and Immunology, Kaohsiung Medical University, Kaohsiung, Taiwan; 5 Department of Marine Biotechnology and Resources, National Sun Yat-sen University, Kaohsiung, Taiwan

## Abstract

Antibiotic-resistant patterns, a *mecA* homologue complex, and staphylococcal cassette chromosome *mec* (SCC*mec*) were analysed in samples of ready-to-eat (RTE) street food in Taiwan. RTE food samples (270) were collected in three densely populated Taiwanese cities between June and November 2014. Among 14 strains being identified as methicillin-resistant coagulase-negative staphylococci (MRCoNS), genetic diversities was determined by PFGE analysis. SCC*mec* types IV, V, VIII and TXG-24 were detected in 9, and *mecA*_*Ss*_ (a *mecA* homologue) detected in 8. The *mecA*_*Ss*_ gene complex from *S. sciuri* subsp. *sciuri* TXG-24 was found to be closely related to those found in both *S. sciuri* subsp*. sciuri* (ATCC29062) and *S. sciuri* subsp*. rodentium* (ATCC700061). SCC*mec*_TXG24_ carries a class A *mec* complex, a *ccrA5B3*-like gene complex, a heavy metal gene complex, and an IS*1216* mobile element carrying *tet*(S). Matching identity to *ccrA5* was 84.5% for *ccrA* in *S. pseudintermedius* KM241. Matching identify to *ccrB3* was 92.1% for *ccrB* in *S. pseudintermedius* AI16. Similar *ccrA* and SCC*mec* boundary sequences suggest that SCC*mec* is easily transmitted to coagulase-negative staphylococci (CoNS). Based on MRCoNS strains identified in this research, Taiwanese RTE food products likely carry multiple antibiotic resistance genes that can be transmitted to hospitals and other clinical settings.

Convenient, cheap and popular, ready-to-eat (RTE) food products in Taiwan are found in public markets, train stations, sidewalk stalls, and many other locations. The microbiological quality of RTE foods has gained attention due to the 4,284 cases of food poisoning that were officially reported between 1991 and 2010, with the number of cases increasing yearly^1^. Accordingly, the literature on the microbiological quality of cold RTE foods (e.g., sandwiches, noodles, and rice balls served at 18 °C or lower) and RTE-related ingredients (e.g., staples, meats, vegetables, and seafood) in Taiwan has grown significantly^2^,^3^. After analysing 164 RTE food samples served at 18 °C, Fang *et al*. reported a 42.7% incidence of psychrotrophic *Pseudomonas spp.*, 75% coliforms, 7.9% *E. coli*, 49.8% *B. cereus* and 17.9% *S. aureus*^2^. According to a separate study conducted by Wei *et al*., RTE-related products stored at room temperature had the highest incidence of bacterial contamination, and RTE foods served by street vendors in traditional markets had the highest bacterial counts^3^. However, a review of extant studies indicates that few efforts have been made to determine antibiotic resistance in RTE foods.

The first case of methicillin resistance in *Staphylococcus aureus* (MRSA) was reported in Great Britain in 1961^4^. The resistance mechanism has been linked to an alternative penicillin-binding protein (either PBP2a or PBP2′) encoded by *mecA* and transmitted via the excision and insertion of a SCC*mec* element^5^,^6^. SCC*mec* elements share two important features: a *mec* gene complex carrying a *mecA* homologue, and specific insertion sites with flanking repeat sequences via the *ccr* gene complex^6^. Recently, several research teams have reported the potential of coagulase-negative staphylococci (CoNS) for transmitting antibiotic-resistant genes^7^,^8^,^9^,^10^,^11^. Tulinski *et al*. found that CoNS strains isolated from pig farms acted as reservoirs for heterogeneous SCC*mec* elements^9^. Kloos *et al*. have described *S. sciuri* as a reservoir for a methicillin-resistant gene^11^, and Ruzauskas *et al*. have reported the cross-sectional prevalence of methicillin-resistant *S. haemolyticus* in companion animals^7^.

It is generally accepted that RTE food products serve as reservoirs for antimicrobial-resistant bacteria, but transmission and resistance mechanisms in Taiwan require further investigation. For this project, we looked at proportions of methicillin-resistant coagulase-negative staphylococci (MRCoNS) found in samples of spring rolls, cold noodles, and fruit platters collected from RTE vendors in the densely inhabited cities of Kaohsiung, Taichung and Taipei, and attempted to determine their antibiotic resistance mechanisms.

## Results

### MRCoNS characterization

We used *dnaJ* gene sequencing to identify bacterial species in 14 MRCoNS strains (Table 1). The dominant bacteria was *S. sciuri* (6/14, 42.9%), including 3 isolates of *S. sciuri* subsp. *rodentium*, 2 isolates of *S. sciuri* subsp. *sciuri*, and 1 isolate of *S. sciuri* subsp. *carnaticus*, followed by *S. saprophyticus* (4/14, 28.7%), *S. haemolyticus* (2/14, 14.4%), *S. lentus* (1/14, 7%), and *S. pasteuri* (1/14, 7%). The most frequent sources were spring rolls (7/14, 50%), cold noodles (5/14, 35.7%), and fruit platters (2/14, 14.3%). Genetic diversity data as determined by PFGE analysis are shown in Table 1 and Supplementary Fig. S1. Only two *S. saprophyticus* isolates (TPE-21 and TPE-32, isolated from cold noodles and spring rolls, respectively) belong to pulsotype IX (Table 1). According to antimicrobial susceptibility test results, all isolates were resistant to 1–5 antimicrobials, a list that included oxacillin (14/14, 100%), levofloxacin (2/14, 14%), erythromycin (10/14, 71.4%), tetracycline (9/14, 64.3%), gentamicin (4/14, 28.7%; 1 of the 4 was gentamicin-intermediate), and vancomycin-intermediate (1/14, 7%) (Table 1).

*mecA* was detected in all 14 oxacillin-resistant isolates (14/14, 100%) (Table 1). Among the 10 erythromycin-resistant isolates, *S. saprophyticus* KHH-2, *S. sciuri* subsp. *sciuri* TXG-24, and *S. sciuri* subsp. *rodentium* TXG-28 carried both *ermA* and *ermC* genes, while *S. haemolyticus* TXG-25, *S. sciuri* subsp. *sciuri* TXG-15, and *S. sciuri* subsp. *rodentium* TPE-18 only carried the *ermC* gene. No *erm* genes were detected in the other 4 erythromycin-resistant isolates. Among the 9 tetracycline-resistant isolates, *S. sciuri* subsp. *sciuri* TXG-15 harboured 3 tetracycline-resistant genes, while *S. haemolyticus* TXG-25 and *S. sciuri* subsp. *rodentium* TXG-28 and TPE-18 contained *tet*(K). Both *tet*(M) and *tet*(K) were found in *S. saprophyticus* KHH-20, and *tet*(M) and *tet*(O) were found in *S. lentus* TXG-26. We also observed *aac*(*6*′)*Ie-aph*(*2*″)*Ia* in all gentamicin-resistant isolates (4/4, 100%) (Table 1). Staphylococcal super-antigenic genes encoded staphylococcal enterotoxins (SEs), an SE-related toxin, and toxic shock syndrome toxin-1 (TSST-1). Among the 14 isolates, 12 (85.7%) carried one or more staphylococcal super-antigenic genes, but they were not detected in *S. haemolyticus* TXG-25 or *S. saprophyticus* TPE-21. Among enterotoxins and enterotoxin-like proteins, the most prevalent genes were *sec* (3/14, 21.4%), *selk* (5/14, 35.7%) and *seln* (5/14, 35.7%). The *sed* and *selp* genes were not detected in any isolates (Table 1).

### Genetic analysis of the *mecA*_*Ss*_ gene complex and SCC*mec*_TXG24_

**Figure 1:**
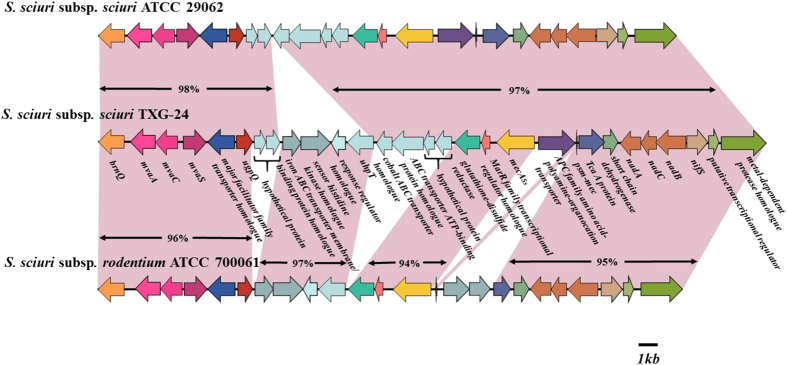
Genomic structure of the *mecA*_*Ss*_ complex in *S. sciuri* subsp. *sciuri* TXG-24. Homologous regions are in pink. Arrows indicate genes and their directions. Most sequences are closely related to the sequences of *S. sciuri* subsp. *sciuri* (ATCC29062) and *S. sciuri* subsp. *rodentium* (ATCC700061).

Gene analysis results indicate the presence of two *mecA* homologues (*mecA* and *mecA*_*Ss*_) in *S. sciuri* subsp*. sciuri* TXG-24. Genomic structure analysis data for the *mecA*_Ss_ region are shown in Fig. 1. The *mecA*_*Ss*_ gene complex of TXG-24 is closely related to *S. sciuri* subsp*. sciuri* ATCC29062 (GenBank accession number AB547234.1), with the exception of a downstream 4-gene cobalt ABC transporter homologue. The *mecA*_*Ss*_ region of *S. sciuri* subsp. *rodentium* ATCC700061 (AB547235.1) shares a high degree of similarity with TXG-24, except for the upstream *ugpQ* of two hypothetical protein genes, the ABC transporter gene, and the amino acid/polyamine/organocation (APC) family transporter gene.

**Figure 2:**
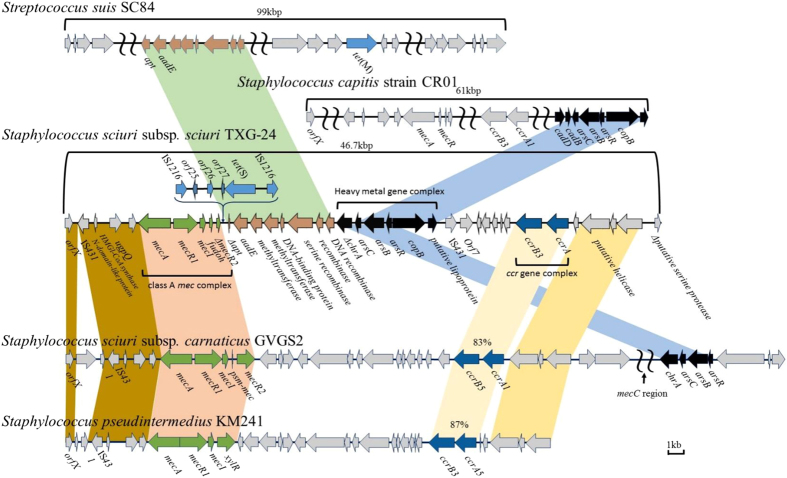
Data from a genomic analysis of SCC*mec* in *S. sciuri* subsp. *sciuri* TXG-24, compared to data for SCC*mec* in *S. sciuri* subsp. *carnaticus* GVGS2, *S. pseudintermedius* KM241, *S. capitis* CR01, and a partial sequence in the integrative and conjugative element (ICE) of *Streptococcus suis* SC84. Colours indicate various homologous regions in bacterial isolates.

The SCC*mec* element of *S. sciuri* subsp*. sciuri* TXG-24 has a complex genomic structure that contains a class A *mec* gene complex (IS*431*-*mecA*-*mecR1*-*mecI*), an IS*1216* mobile element carrying *tet*(S), partial DNA recombinase with methyltransferase, a heavy metal-resistant gene complex, and a *ccr* gene complex (Fig. 2). The *mec* gene complex of SCC*mec*_TXG24_ is closely related to *S. sciuri* subsp*. carnaticus* GVGS2 (HG515014) and *S. pseudintermedius* KM241 (AM904731), except for two hypothetical protein genes and a truncated *mecR2* gene. The SCC*mec*_TXG24_ region containing partial DNA recombinase with methyltransferase is highly similar to the comparative region of *Streptococcus suis* SC84 (FM252031.1), except for a truncated *apt* gene. Compared to *S. capitis* CR01 (KF049201), the heavy metal-resistant gene complex is associated with the absence of two cadmium-resistant genes (*cadD* and *cadB*). The proximal left boundary of SCC*mec* consists of the *ccr* gene complex, the putative helicase gene, and some hypothetical protein genes that are associated with comparative regions in *S. sciuri* subsp*. carnaticus* GVGS2 and *S. pseudintermedius* KM241.

### Analysis of insertion sequence element carrying the tet(S) tetracycline-resistant gene

**Figure 3:**
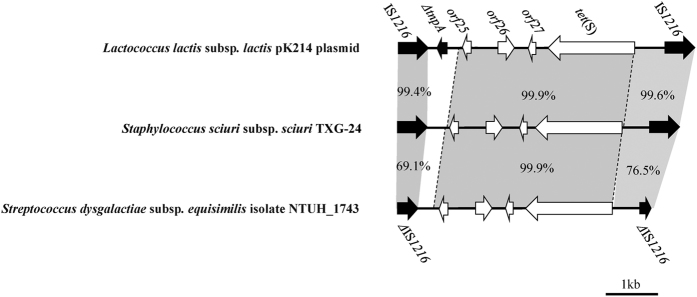
Data from a genetic analysis of the tetracycline-resistant *tet*(S) gene complex inserted in SCC*mec*TXG24 and compared to the complexes *Lactococcus lactis* subsp. *lactis* plasmid pK214 and *Streptococcus dysgalactiae* subsp. *equisimilis* NTUH_1743. Homologous regions are shaded in gray. Black arrow, transposase. Corresponding regions are shadowed.

The *tet*(S)-carrying IS*1216* mobile element was found at the 3′ end of *ΔmecR2* (Fig. 2). According to our sequence analysis, *orf25*-*orf26*-*orf27*-*tet*(S) had a high degree of similarity with both the *Lactococcus lactis* subsp*. lactis* pK214 plasmid (GenBank accession number X92946) and *Streptococcus dysgalactiae* subsp*. equisimilis* NTUH_1743 (EF682209) (Fig. 3). Comparisons of IS*1216* regions revealed exceptionally high degrees of shared identity (99.4% and 99.6%) with the *L. lactis sp. lactis* pK214 plasmid, but much lower degrees of shared identity (69.1% and 76.5%) with *S. dysgalactiae* subsp*. equisimilis* NTUH_1743 due to a truncated gene. The *ΔtnpA* gene was only found downstream of *orf25* in *L. lactis sp. lactis* pK214.

### *ccr* gene phylogenetic trees

**Figure 4:**
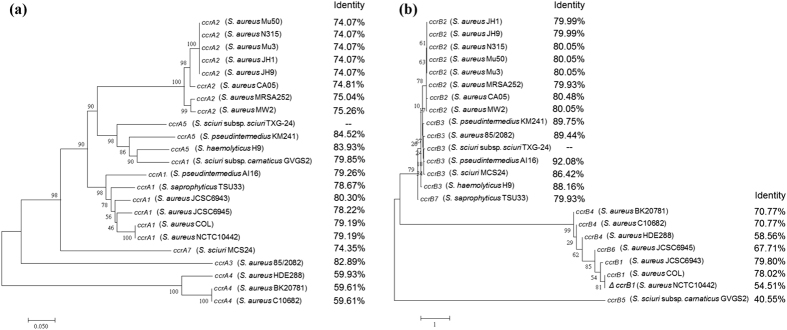
Phylogenetic trees for the cassette chromosome recombinase (*ccr*) gene. (**a**) 23 *ccrA* genes. (**b**) 23 *ccrB* genes. Trees were generated using neighbour-joining MEGA7 software. Numbers next to nodes indicate confidence levels, expressed as percentages of occurrence over 2,000 bootstrap samples. Scale bar indicates evolutionary distance.

SCC*mec* is a genetic element that encodes methicillin resistance and that carries a unique site-specific recombinase (the *ccr* gene) in charge of SCC*mec* element integration and excision^6^,^12^. For the present study, we identified a *ccr* gene complex in *S. sciuri* subsp*. sciuri* TXG-24. Lengths of *ccrA* and *ccrB* were 1350 and 1629 bp, respectively. Phylogenetic trees for the *ccrA* and *ccrB* sequences (23 each) are shown in Fig. 4a and b. *ccrA* matching identity was 84.5% to *ccrA5* in *S. pseudintermedius* KM241 (GenBank accession number AM904731). *ccrB* matching identity was 92.1% to *ccrB3* in *S. pseudintermedius* AI16 (LN864705.1).

### SCC*mec*_TXG24_ boundaries

**Figure 5:**
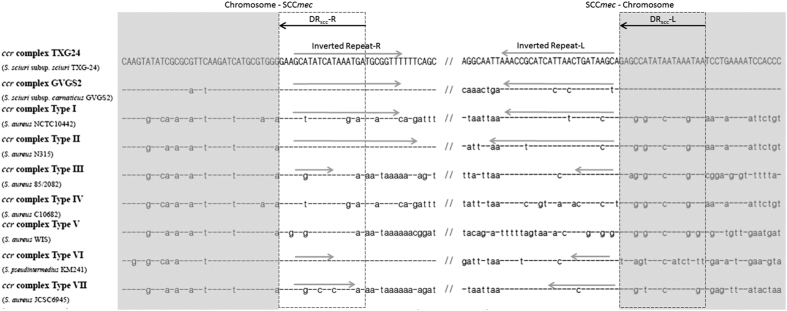
SCC*mec* boundaries. Left and right boundaries of SCC*mec* types I to VII and the SCC*mec* element of *S. sciuri* subsp. *carnaticus* GVGS2 were aligned with SCC*mec*TXG24. Black arrows indicate direct repeats (DRs). Gray arrows indicate inverted repeats (IRs) of SCC*mec* elements.

To investigate SCC*mec*_TXG24_ boundaries, we aligned the left and right boundaries of SCC*mec* types I–VII with the SCC*mec* element of *S. sciuri* subsp*. carnaticus* GVGS2 (Fig. 5). SCC*mec*_TXG24_ integration occurred at almost the same nucleotide position at the 3′ end of the *orfX* gene as the SCC*mec* complex of *S. sciuri* subsp*. carnaticus* GVGS2 and *S. pseudintermedius* KM241, with both sharing identical direct repeats (DR) at their left and right boundaries. However, nucleotide positions in the other SCC*mec* types were different from that of SCC*mec*_TXG24_, and the inverted repeats (IR) of each SCC*mec* type were variant.

### SCC*mec* typing and *mecA*_*Ss*_ detection in 14 MRCoNS strains

To investigate SCC*mec* distribution in Taiwan, we analysed 14 strains of MRCoNS from the 270 RTE food samples. Four SCC*mec* types (IV, V, VIII and TXG-24) were identified in 9 strains (9/14, 64.3%); the other 5 were non-typeable (Table 2). The dominant form was SCC*mec* type VIII (3/9, 33.3%), found in *S. sciuri* subsp. *carnaticus* KHH-57 and TPE-33, and in *S. lentus* TXG-26. SCC*mec* type IV (2/9, 22.2%) was found in *S. pasteuri* TPE-12 and *S. saprophyticus* TPE-32. SCC*mec* type V (2/9, 22.2%) was found in *S. haemolyticus* KHH-11 and *S. sciuri* subsp. *rodentium* TXG-28. SCC*mec*_TXG24_ (2/9, 22.3%) was found in *S. sciuri* subsp. *sciuri* TXG-24 and *S. sciuri* subsp. *rodentium* TPE-18. The intrinsic *mecA*_*Ss*_ gene was present in 8 of the 14 MRCoNS strains (57.1%).

## Discussion

In their study of five types of RTE food products in Taiwan, Fang *et al*. reported 75%, 49.8%, 42.7%, 17.9% and 7.9% contamination rates for coliform, *Bacillus cereus, Pseudomonas* spp., *S. aureus* and *E. coli*, respectively, in food products stored at 18 °C^2^. *S. aureus* was found in 26.1% of all ham samples, 21.4% of all seafood samples, 15.4% of other meat samples, and 13.6% of all vegetable samples. A separate study conducted in southern Taiwan found a 9.5% incidence of *S. aureus* contamination in RTE food products purchased from warehouse stores, 12.7% from traditional markets, and 19.0% from supermarkets^3^. The two research teams reported the presence of different pathogens in RTE food, but did not address antimicrobial susceptibility or resistance pattern tendencies. For the present study, we isolated 14 MRCoNS strains that were resistant to at least one antibiotic, and identified the dominant sources as spring rolls filled with salad ingredients and stewed ground pork wrapped in thin pastry dough, both prepared by glove-wearing vendors (Table 1). The fillings and pastry cracks are likely bacteria reservoirs^13^. The second most common source was cold noodles mixed with some kind of sauce, with bacterial proliferation likely due to the relatively higher pH value of the sauce or improper storage temperature^2^. Bacterial contamination of fruit platters (the third most common source) was likely due to the improper cleaning of knives. Regardless of actual cause or transmission route, the data indicate that RTE food contamination is a likely avenue for transmitting antibiotic-resistant genes and food-borne diseases^14^,^15^,^16^.

Determining genetic relationships in bacterial isolates is an important task for monitoring the spread of bacteria. In one study conducted in Turkey, genetic diversity data for 154 multi-drug-resistant strains of *S. aureus* found in 1,070 RTE food samples suggested multiple routes for various isolates^17^. In the present study, only two *S. saprophyticus* isolates (TPE-21 and TPE-32, both from Taipei city) shared the same pulsotype, indicating genetic diversity in our RTE food samples (Supplementary Fig. S1).

Staphylococcal enterotoxin (SE) contaminated food have been reported in foodborne illness^18^. Fijałkowski *et al*. reported that the prevalence of toxin genes in 75 different staphylococcal isolates from 41 food samples in Poland^19^. The most prevalent SE genes were *sei* (27/75, 36%), followed by *seln* (24/75, 32%) and *sed* (23/75, 31%). Chiang *et al*. reported that 109 (74.1%) *S. aureus* isolates contained one or more SE genes in Taiwan^20^. The most detected SE genes were *sei* (45/147, 30.6%), followed by *sea* (42/147, 28.6%) and *seb* (30/147, 20.4%). These studies and our finding revealed that SEs production of staphylococcal isolates may be associated with food poisoning^19^,^20^. Our study found that the dominant SE genes were *selk* (5/14, 35.7%) and *seln* (5/14, 35.7%), followed by *sec* (3/14, 21.4%) (Table 1). Further studies are warranted to determine the importance of SEs-producing CoNS in RTE food.

To date, the *mecA* gene has been found in multiple homologues, including *mecA1* (*mecA1, mecA*_*Ss*_ and *mecA*_*Sv*_)^21^,^22^, *mecA*_*Sf*_^22^, and *mecC* (formerly *mecA*_*LGA251*_)^23^. Although *mecA*_*Ss*_ (from *S. sciuri*) and *mecA*_*Sv*_ (from *S. vitulinus*) share 80% and 91% identities with *mecA*, respectively, neither gene is associated with oxacillin resistance^22^. *mecA*_*Sf*_ (from *S. fleurettii*), which belongs to the class A *mec* complex, shares 99% identity with the *mecA* gene, suggesting that *S. fleurettii* may be the ancestor of the SCC*mec* element in MRSA^22^. We found a close relationship between the TXG-24 *mecA*_*Ss*_ gene complex and a comparative region of *S. sciuri* subsp*. sciuri* ATCC29062 (GenBank accession number AB547234.1) that is not associated with oxacillin resistance (Fig. 1).

The CoNS-acquired *mecA* gene, which has been the focus of multiple studies, is a likely reservoir for transmitting antibiotic-resistant genes^7^,^8^,^9^,^10^,^11^. Of the 14 MRCoNS strains that we tested, the most prevalent was *S. sciuri*—a widespread *Staphylococcus* species among animals and humans. Reported in a wide range of food products, this bacteria has been described as a reservoir for the methicillin-resistant gene^11^,^24^,^25^. *S. sciuri* was first described by Kloos *et al*. and originally isolated from both human and animal skins^26^. According to one study, *ccr* genetic diversity in methicillin-susceptible *S. sciuri* may be useful for capturing the *mecA* gene and assembling the SCC*mec* element^27^. Two research teams have shown that *S. saprophyticus, S. haemolyticus* and *S. lentus* in RTE foods are likely routes for antibiotic-resistant gene transmission in Poland^28^,^29^. Specifically, Podkowik *et al*. reported that 40% (17/42) of the CoNS strains they examined were resistant to 4 or more antibiotics, especially 15 isolates (36%) harbouring the *mecA* gene^28^. Chajecka-Wierzchowska *et al*. found that 56.9% (33/58) of the CoNS strains they tested were resistant to at least one antibiotic, with 24 isolates (41.3%) harbouring the *mecA* gene^29^. In Taiwan, SCC*mec* types IV and V have been described as prevalent in community-associated MRSA; these same SCC*mec* types were also found in the MRCoNS strains we analysed for the present study (Table 2)^30^,^31^. Combined, these data indicate that MRCoNS strains can serve as reservoirs for transmitting the SCC*mec* element to and from MRSA.

Many *ccr* gene complexes have been identified in MRSA^23^,^32^,^33^. Multiple *ccr* variants have been found in CoNS strains—for example, *ccrA5B3* in *S. pseudintermedius* KM241 and both *ccrA5B13* and *ccrA5B9* in *S. sciuri*^27^,^34^. Different compositions of the *ccr* gene complex may be due to dissimilarities in recognised insertion sites^35^,^36^. We found similar *ccrA* boundaries sequences in *S. sciuri* subsp. *sciuri* TXG-24, *S. pseudintermedius* KM241, and *S. sciuri* subsp. *carnaticus* GVGS2, suggesting that SCC*mec* is easily transmitted across these and perhaps other *Staphylococcus* species (Figs 4a and 5).

In summary, we found that CoNS strains in contaminated RTE food samples collected in three Taiwanese cities were resistant to multiple types of antibiotics; it is likely that the associated antibiotic-resistant genes can be easily transmitted to other food products, to the homes of consumers, and to hospitals and other clinics. Since *S. sciuri* carries diverse *ccr* genes that are globally distributed, further research is called for to determine or refute its role as a reservoir for antibiotic-resistant gene transmission.

## Methods

### Sample collection and microbiological analysis

A total of 270 food samples (90 spring rolls, 90 cold noodle bowls and 90 fruit platters) were collected between June and November of 2014. All samples were randomly procured and transported to our laboratory in their original packaging, either within 1 h at the original temperature (Kaohsiung and Taichung samples) or 2 h refrigerated at 4 °C (Taipei samples).

For each sample, 10 g were homogenised using a stomacher sample blender, and enriched in brain-heart infusion broth (BD Biosciences) overnight at 37 °C. Single loopfuls of each bacterial suspension were plated on mannitol salt agar. Single colonies were placed on Muller-Hinton agar with 2% NaCl and 4 μg/ml oxacillin. Bacterial identification was performed by *dnaJ* gene sequencing as previously described^37^.

### Antimicrobial susceptibility testing

Antimicrobial susceptibility testing was performed using standard agar dilution methods according to Clinical and Laboratory Standards Institute guidelines^38^. Minimum inhibitory concentration (MIC) was defined as the lowest concentration of antibiotic preventing bacterial growth after 16–20 h of incubation at 37 °C. The following antimicrobial agents were tested: erythromycin, gentamicin, levofloxacin, oxacillin, tetracycline and vancomycin.

### Pulsed-field gel electrophoresis (PFGE)

PFGE typing of *Sma*I-digested DNA (New England BioLabs, Ipswich, MA) was performed as previously described^39^. Electricity (200 volts) was applied for 20 h at 13 °C, with pulse durations ranging from 5.3 to 34.9 sec at 6 V/cm. Dice similarity indices^40^ were used to construct pulsotype relationship dendrograms using an unweighted pair group method with arithmetic means. Pulsotypes exhibiting 85% similarity were assigned to the same clusters.

### PCR detection of antibiotic-resistant genes and staphylococcal enterotoxin genes

PCR was used to detect the presence of the following antibiotic-resistant genes: gentamicin (*aac*(*6*′)*Ie-aph*(*2*″)*Ia*), oxacillin (*mecA*), vancomycin (*vanA, vanB*), erythromycin (*ermA, ermB, ermC*), and tetracycline (*tet*(M), *tet*(O), *tet*(K)). Primer sets were selected based on a previous study^41^. The presence of staphylococcal enterotoxin genes, *sea, seb, sec, sed, see, seg, seh, sei, selj, selk, sell, selm, seln, selo, selp, selq, selr* and *tst1*, were determined by PCR using primer sets from a previous study^42^.

### Identification of SCC*mec*_TXG24_ and the *mecA*_*Ss*_ gene complex

Genomic DNA from *S. sciuri* subsp. *sciuri* TXG-24 was extracted manually. Total DNA was subjected to quality control using agarose gel electrophoresis and quantified by Qubit (Invitrogen, Thermo Fisher Scientific, Waltham, MA). The *S. sciuri* subsp. *sciuri* TXG-24 genome was sequenced using massively parallel sequencing Illumina (San Diego, CA). Two DNA libraries were constructed: a paired-end library with a 500 bp insert, and a mate-pair library with a 5 kb insert. Both libraries were sequenced with the HiSeq2500 ultra-high-throughput sequencing system (Illumina, San Diego, CA) (PE125 strategy). Library construction and sequencing was performed at Beijing Novogene Bioinformatics Technology Co., Ltd. An in-house quality control program was used for both paired-end and mate-pair reads. Illumina PCR adapter reads and low quality reads were filtered and assembled with SOAPdenovo^43^,^44^ to generate scaffolds. All reads were used for subsequent gap closures. SCC*mec*_TXG24_ and *mecA*_*Ss*_ gene complex nucleotide sequences from *S. sciuri* subsp. *sciuri* TXG-24 were added to GenBank (accession numbers KX774481 and KX774480, respectively).

### Phylogenetic tree analysis

The *ccrA* and *ccrB* genes identified in this work were compared with 22 publicly available *Staphylococcus* spp. sequences: *S. aureus* strains JCSC6943, JCSC6945, COL, NCTC10442, CA05, JH1, JH9, MRSA252, Mu3, Mu50, MW2, N315, 85/2082, BK20781, C10682 and HDE288 (GenBank accession numbers AB505628.1, AB505630.1, CP000046, AB033763.2, AB063172.2, CP000736, CP000703, BX571856, AP009324, BA000017, BA000033, BA000018, AB037671.1, FJ670542.1, FJ390057.1, and AF411935.3, respectively); *S. pseudintermedius* strains KM241 and AI16 (AM904731 and LN864705.1); *S. haemolyticus* H9 (EU934095); *S. saprophyticus* subsp. *saprophyticus* TSU33 (AB353724.1); *S. sciuri* MCS24 (AB587080.1); and *S. sciuri* subsp. *carnaticus* GVGS2 (HG515014). Phylogenetic trees were analysed by MEGA7 using the neighbour-joining method; tree topologies were estimated using bootstrap analyses with 2,000 replicates to achieve confidence intervals as indicated on each tree node^45^. Identities shown after each *ccr* gene were aligned and calculated using DNAman (Lynnon Biosoft, Quebec).

### SCC*mec* type determination and *mecA*_*Ss*_ gene detection

SCC*mec* types were determined by *mec* and *ccr* gene complexes as described in our previous study^39^. SCC*mec*_TXG24_ was determined by the class A *mec* complex and *ccr* gene (*ccrA5B3*) (Supplementary Table S1), and *mecAs* was determined by mecAs-F and mecAs-R (Supplementary Table S1).

## Additional Information

**How to cite this article:** Yang, T.-Y. *et al*. *mecA*-related structure in methicillin-resistant coagulase-negative staphylococci from street food in Taiwan. *Sci. Rep.* **7**, 42205; doi: 10.1038/srep42205 (2017).

**Publisher's note:** Springer Nature remains neutral with regard to jurisdictional claims in published maps and institutional affiliations.

## Supplementary Material

Supplementary Table & Figure

## Figures and Tables

**Table 1:** Antibiotic resistance and pulsotypes identified in CoNS isolated from the 270 RTE food samples.

Area	Species and Strain	Isolated Source	Pulsotype	Antibiotic Resistance Phenotype	Antibiotic Resistance Genotype	*S. aureus* Super Antigenic Toxin Genotype
Kaohsiung						
	*S. saprophyticus* KHH-2	cold noodles	X	OXA, ERY	*mecA, ermA, ermC*	*seg, seh, selo*
	*S. haemolyticus* KHH-11	fruit platter	III	OXA, ERY	*mecA*	*sec, seh, selj*
	*S. saprophyticus* KHH-20	spring roll	XI	OXA, TET, ERY	*mecA, tet*(M), *tet*(K)	*selk, seln*
	*S. sciuri* subsp. *carnaticus* KHH-57	spring roll	XII	OXA, TET, ERY	*mecA*	*sea, selk, seln*
Taichung						
	*S. sciuri* subsp. *sciuri* TXG-15	cold noodles	IV	OXA, TET, ERY	*mecA, ermC, tet*(M), *tet*(O), *tet*(K)	*sec, selj, selk, seln*
	*S. sciuri* subsp. *sciuri* TXG-24	spring roll	VI	OXA, VAN^a^, TET, ERY	*mecA, ermA, ermC*	*seb, selk, seln*
	*S. haemolyticus* TXG-25	cold noodles	XIII	OXA, TET, GEN, LVX, ERY	*mecA, ermC, tet*(K), *aac*(*6*′)*Ie-aph*(*2*″)*Ia*	ND^b^
	*S. lentus* TXG-26	spring roll	I	OXA, TET, GEN	*mecA, tet*(M), *tet*(O), *aac*(*6*′)*Ie-aph*(*2*″)*Ia*	*sei, selr*
	*S. sciuri* subsp. *rodentium* TXG-28	spring roll	II	OXA, TET, GEN, LVX, ERY	*mecA, ermA, ermC, tet*(K)*. aac*(*6*′)*Ie-aph*(*2*″)*Ia*	*selk, seln*
Taipei						
	*S. pasteuri* TPE-12	fruit platter	VII	OXA, GEN^a^, ERY	*mecA, aac*(*6*′)*Ie-aph*(*2*″)*Ia*	*see, selm*
	*S. sciuri* subsp. *rodentium* TPE-18	cold noodles	VIII	OXA, TET, ERY	*mecA, ermC, tet*(K)	*sea*
	*S. saprophyticus* TPE-21	cold noodles	IX	OXA	*mecA*	ND^b^
	*S. saprophyticus* TPE-32	spring roll	IX	OXA	*mecA*	*sell, selq, tst1*
	*S. sciuri* subsp. *rodentium* TPE-33	spring roll	V	OXA, TET	*mecA*	*sec*

Abbreviations: OXA, oxacillin; ERY, erythromycin; TET, tetracycline; VAN, vancomycin; GEN, gentamicin; LVX, levofloxacin. ^a^Intermediate resistance to antibiotic. ^b^ND, not detected.

**Table 2:** SCC*mec* types and *mecA*_*Ss*_ in 14 MRCoNS.

Strain	*mecA*_*Ss*_	*mecA*	*mec* Complex Class	*ccr* Gene	SCC*mec* Type
*S. saprophyticus* KHH-2	**−**	**+**	NT^a^	A4B4	NT
*S. haemolyticus* KHH-11	**−**	**+**	C2	C1	V
*S. saprophyticus* KHH-20	**+**	**+**	A	NT	NT
*S. sciuri* subsp. *carnaticus* KHH-57	**+**	**+**	A	A1B1, A4B4	VIII
*S. sciuri* subsp. *sciuri* TXG-15	**+**	**+**	NT	A1B1, A4B4, A5B3, C1	NT
*S. sciuri* subsp. *sciuri* TXG-24	**+**	**+**	A	A5B3	TXG-24
*S. haemolyticus* TXG-25	**−**	**+**	A	C1	NT
*S. lentus* TXG-26	**+**	**+**	A	A1B1, A4B4	VIII
*S. sciuri* subsp. *rodentium* TXG-28	**−**	**+**	C2	C1	V
*S. pasteuri* TPE-12	**−**	**+**	B	A2B2, A5B3	IV
*S. sciuri* subsp. *rodentium* TPE-18	**+**	**+**	A	A5B3	TXG-24
*S. saprophyticus* TPE-21	**+**	**+**	NT	NT	NT
*S. saprophyticus* TPE-32	**−**	**+**	B	A2B2, C1	IV
*S. sciuri* subsp. *rodentium* TPE-33	**+**	**+**	A	A4B4	VIII

^a^NT, non-typeable.
