# Supplementary material for: mecA-related structure in methicillin-resistant coagulase-negative staphylococci from street food in Taiwan
Source: Sci Rep. 2017 Feb 9;7:42205. doi: 10.1038/srep42205 (PMC5299846; doi:10.1038/srep42205)
Supplement: Supplementary Table & Figure [file srep42205-s1.pdf]

mecA-related structure in methicillin-resistant coagulase-negative staphylococci from street food in Taiwan

Tsung-Ying Yang[1], Wei-Wen Hung[2, 3], Lin Lin[4], Wei-Chun Hung[5], and Sung-Pin Tseng[1, 6*]

[1]Department of Medical Laboratory Science and Biotechnology, College of Health Sciences, Kaohsiung Medical University, Kaohsiung, Taiwan;

[2]Division of Endocrinology and Metabolism, [3]Department of Internal Medicine, Kaohsiung Medical University Hospital, Kaohsiung Medical University, Kaohsiung, Taiwan;

[4] Department of Culinary Art, I-Shou University, Kaohsiung, Taiwan;

[5]Department of Microbiology and Immunology, Kaohsiung Medical University, Kaohsiung, Taiwan;

[6] Department of Marine Biotechnology and Resources, National Sun Yat-sen University, Kaohsiung, Taiwan.

[*]**Corresponding author:** Sung-Pin Tseng

100, Shih-Chuan 1st Road, Kaohsiung, Taiwan

Department of Medical Laboratory Science and Biotechnology, Kaohsiung Medical University

Phone: (886)-7-3121101 ext. 2353, Fax: (886)-7-3113449

Email: tsengsp@kmu.edu.tw

Supplementary Table S1. Primers used in bacterial identification, detection of *mecA*$_{Ss}$ and SCC*mec* typing.

Primer	Nucleotide sequence (5'→3')	Constructed on:	Detection of gene (s)	Amplicon (bp)	Reference
SA-F	GCC AAA AGA GAC TAT TAT GA	*dnaJ*	*dnaJ*	920	12
SA-R	ATT GYT TAC CYG TTT GTG TAC C				
mecAs-F	CGG TAT AAG CAC CAC CAC CA	*mecA*$_{Ss}$	*mecA*$_{Ss}$	687	This work
mecAs-R	ACA ATG ATT ACA ACC ACG ATG GC	APC family transporter			
**<u>SCC*mec* typing:</u>**					
mA1	TGC TAT CCA CCC TCA AAC AGG	*mecA*	*mecA*	286	5
mA2	AAC GTT GTA ACC ACC CCA AGA				
mecI-F	AAT GGC GAA AAA GCA CAA CA	*mecI* upstream -59~-40	*mecI*	481	14
mecI-R	GAC TTG ATT GTT TCC TCT GTT	*mecI* downstream 50~30			
mecRA1	GTC TCC ACG TTA ATT CCA T	*mecR1*	*ΔmecR1*-IS*1272*	1287	14
mDA2	GAT GTC TGT CGA GGA CTC	IS*1272*			
mA7	ATA TAC CAA ACC CGA CAA CTA CA	*mecA*	*mecA*-IS*431* (mA7-IS2)	861	14
IS2 (iS-2)	TGA GGT TAT TCA GAT ATT TCG ATG T	IS*431*			
**<u>*ccr* complex detection:</u>**					
α1	AAC CTA TAT CAT CAA TCA GTA CG	*ccrA1*	*ccrA1B1* (βc-α1)	695	14
α2	TAA AGG CAT CAA TGC ACA AAC ACT	*ccrA2*	*ccrA2B2* (βc-α2)	937	
α3	AGC TCA AAA GCA AGC AAT AGA AT	*ccrA3*	*ccrA3B3* (βc-α3)	1791	
βc	ATT GCC TTG ATA ATA GCC **I**TC T	*ccrB1, ccrB2, ccrB3*			
α4.2	GTA TCA ATG CAC CAG AAC TT	*ccrA4*	*ccrA4B4* (α4.2-β4.2)	1287	14
β4.2	TTG CGA CTC TCT TGG CGT TT	*ccrB4*			
γF	CGT CTA TTA CAA GAT GTT AAG GAT AAT	*ccrC*	*ccrC* (γF-γR)	518	14
γR	CCT TTA TAG ACT GGA TTA TTC AAA ATA T	*ccrC*			
ccrTP-F	AAG CGC AAA ACC ATA TCC TCA	*ccrA5*	*ccrA5B3* complex	1019	This work
ccrTP-R	AGG AGG GTG TTT ACC ATC AGC	*ccrB3*			

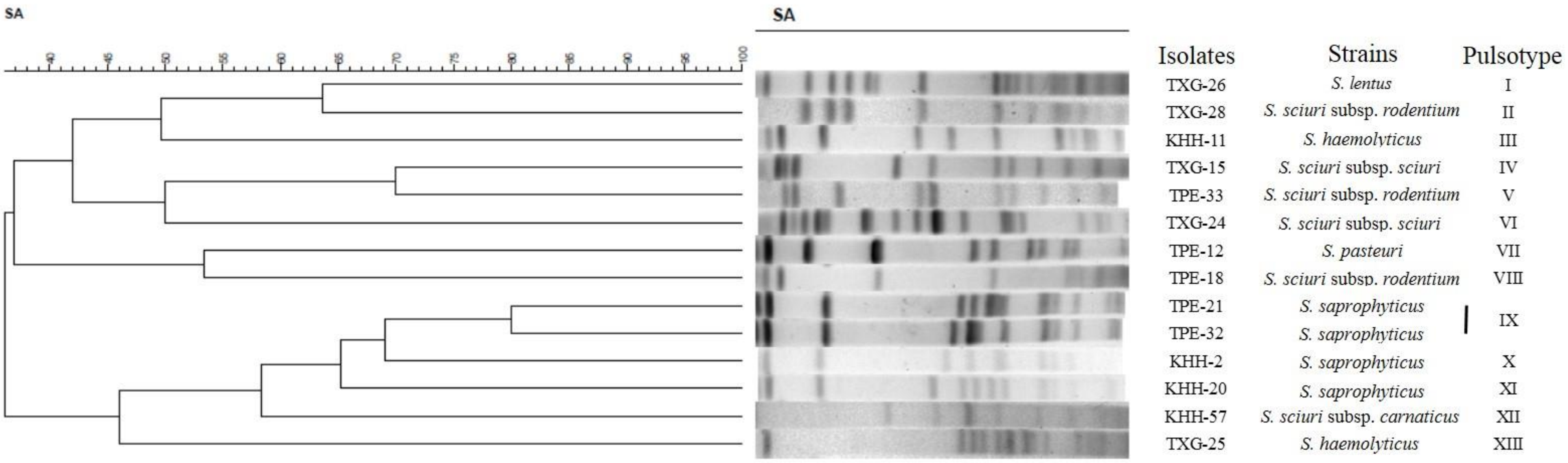

Supplementary Figure S1. Dendrogram of pulsotype relationships developed through the unweighted pair-group method with arithmetic mean (UPGMA) using BioNumerics v.6.5 (Applied Maths, Sint-Martens-Latem, Belgium). Pulsotypes were assigned to the same clusters if they exhibited 80% similarity in the dendrogram.
